# Activation of autophagy protects against cholestasis-induced hepatic injury

**DOI:** 10.1186/2045-3701-4-47

**Published:** 2014-08-26

**Authors:** Lu Gao, Gang Lv, Xianling Guo, Yingying Jing, Zhipeng Han, Shanshan Zhang, Kai Sun, Rong Li, Yang Yang, Lixin Wei

**Affiliations:** Tumor Immunology and Gene Therapy Center, Eastern Hepatobiliary Surgery Hospital, The Second Military Medical University, 225 Changhai Road, Shanghai, 200438 China; Department of Hepatic Surgery, Eastern Hepatobiliary Surgery Hospital, The Second Military Medical University, Shanghai, China; Central laboratory, Renji hospital, Shanghai Jiaotong University School of Medicine, Shanghai, China

**Keywords:** Autophagy, Bile acid, Cholestasis, Hepatocyte, Reactive oxygen species

## Abstract

**Background:**

Cholestasis is characterized by an abnormal accumulation of bile acids and causes hepatocellular injury. Recent studies show that autophagy is involved in the pathophysiology of many liver diseases. The potential role of autophagy in preventing cholestatic hepatotoxicity, however, has rarely been investigated. The aim of this study was to examine whether autophagy is involved in the cholestatic hepatotoxicity.

**Results:**

We found that bile duct ligation (BDL) led to cholestatic liver injury and hepatocytic autophagy activation in the mice. Suppression of autophagy with Chloroquine (CQ) increased liver injury and hepatocytes apoptosis; while activation of autophagy by rapamycin reduced cholestasis hepatotoxicity. In L02 normal liver cells, Glycochenodeoxycholate (GCDC) treatment would induce autophagy. Inhibition of autophagy by CQ could promote GCDC-induced cell apoptosis. In contrast, rapamycin treatment could protect against GCDC-induced cell death. Furthermore, autophagy contributed to the liver cells survival via modulation of reactive oxygen species (ROS).

**Conclusions:**

These findings indicate that autophagy protects against cholestasis induced liver injury and hepatocyte apoptosis by eliminating ROS accumulation. Our data suggest that enhancement of autophagy may be a therapeutic strategy to mitigate cholestatic liver injury.

## Introduction

Cholestasis is a commonly clinical pathology of many diseases, which is characterized by an abnormal accumulation of bile acids. Many liver diseases have been demonstrated to have cholestatic pathophysiology, such as cholangiocarcinoma, bile duct stone, primary biliary cirrhosis, biliary atresia, and primary sclerosing cholangitis [1–3]. Initial studies suggested that the accumulation of hydrophobic bile acids in the liver contributes to cholestasis associated liver injury [4], and that Glycochenodeoxycholate (GCDC), the main toxic component of bile acid in patients, could induce necrosis in freshly isolated hepatocytes and primary cultured hepatocytes [5, 6], or induce apoptosis in isolated liver cell models [7, 8]. In recent years, the possible molecular mechanisms of liver damage induced by cholestasis, including oxidative stress, mitochondrial damage, and cell membrane disruption through their detergent action on lipid components have been found in bile acid-induced hepatocyte death. Despite this observation, the definitive mechanisms that underlie the hepatic injury during cholestatic liver diseases remain incompletely understood.

Autophagy is a catabolic process that enables cells to recycle amino acids and other intracellular nutrients to obtain energy [9]. It is believed to be a mechanism for selective elimination of protein and dysfunctional organelles that are damaged under pathological conditions [10]. Autophagy dysfunction is associated with various diseases, such as cancer, neurodegeneration, gastrointestinal disorders, microbial infection, and aging [11]. The genes that regulate autophagy were first identified in yeasts. Among them, LC3, a marker of autophagosomes in mammalian cells, is activated and then relocalizes to intracellular vesicles when the lipid bilayer structure sequesters cytoplasm to form autophagosmes [12]. In addition, p62 is a multifunctional protein that binds to LC3 and to ubiquitinated proteins, which mediates the recognition of protein aggregates for autophagic clearance [13]. The accumulation of p62 marks the dysfunctional autophagy which is not enough to process the damaged proteins bound to p62 [14]. In recent years, various studies reported that autophagy actively participates in liver physiology and pathogenesis [15, 16]. In liver, it was found that autophagy plays important roles in cytoprotection against multiple pathological insults, including liver steatosis, liver injury, dyslipidemia in alcoholic, and non-alcoholic fatty liver conditions [17–20]. However, whether autophagy plays roles in cholestasis is unclear.

In this study, we investigated whether the autophagy machinery could be activated in liver injury induced by cholestasis; we hypothesized that autophagy contributes to the cell survival via reactive oxygen species (ROS) modulation in hepatocytes.

## Results

### Bile duct ligation promoted autophagy in the mouse liver

**Figure 1:**
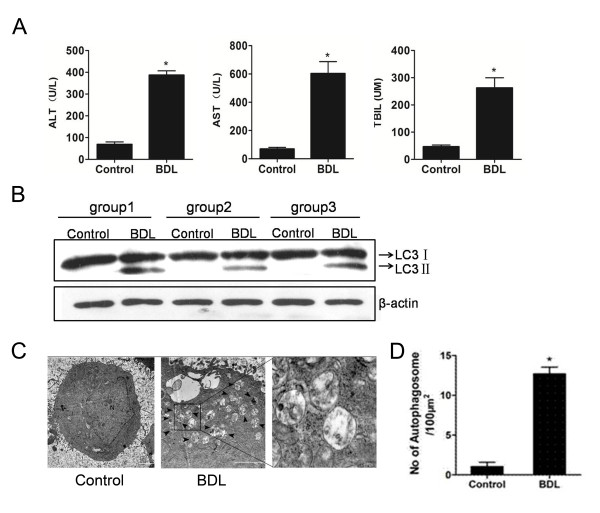
**Bile duct ligation leads to liver injury and activation of autophagy. (A)** Plasma ALT, AST and TBIL levels were analyzed in mice treated with either BDL or control after 2 weeks. Data were expressed as mean ± SD. (*: P < 0.05). **(B)** Representative liver sections were collected from the mice of BDL and control group, then protein levels of LC3 of liver samples were analyzed by an immunoblot assay. **(C)** The liver samples were processed for electron microscope and representative electron micrographs were shown. Arrows denote autophagosomes. (N: nucleus; Bar: 20 µm). **(D)** Data were derived from three independent experiments. (*: P < 0.05).

In this part, we carried out a bile duct ligation (BDL) experiment to mimic the pathophysiogical process of cholestasis in *vivo*. Mice were euthanized after 2 weeks post-BDL or sham surgery. Blood was collected and ALT and AST activities were quantified. As shown in Figure 1A, Both ALT, AST and TBIL levels were significantly elevated in BDL group, indicating that BDL led to hepatic injury and cholestasis accumulation in mice liver. In the subsequent research, we observed the changes in autophagy after treatment with BDL. Since autophagy is generally considered as a protective mechanism in response to stresses, such as starvation, hypoxia and DNA damage, we detected the impact of BDL on autophagy level of liver cells in mice. LC3 is a special marker for indicative of autophagosomal activation. Detection of the conversion from LC3-I (cytosolic form) to LC3-II (membrane-bound lipidated form) by immunoblotting is considered as a hall mark of autophagic activity [21]. Through the blot of LC3, the expression of LC3-II level was markedly increased in BDL group, whereas that of the control group was weak (Figure 1B). Furthermore, electron microscope also showed an apparent double-membrane vacuolar structures with feature of autophagosome following treatment of BDL, but the number of autophagosome was rarely detected in the livers of the control group (Figure 1C and D). These results suggest that bile duct ligation leads to liver injury and activation of autophagy.

### GCDC induced autophagy in L02 hepatocytes

**Figure 2:**
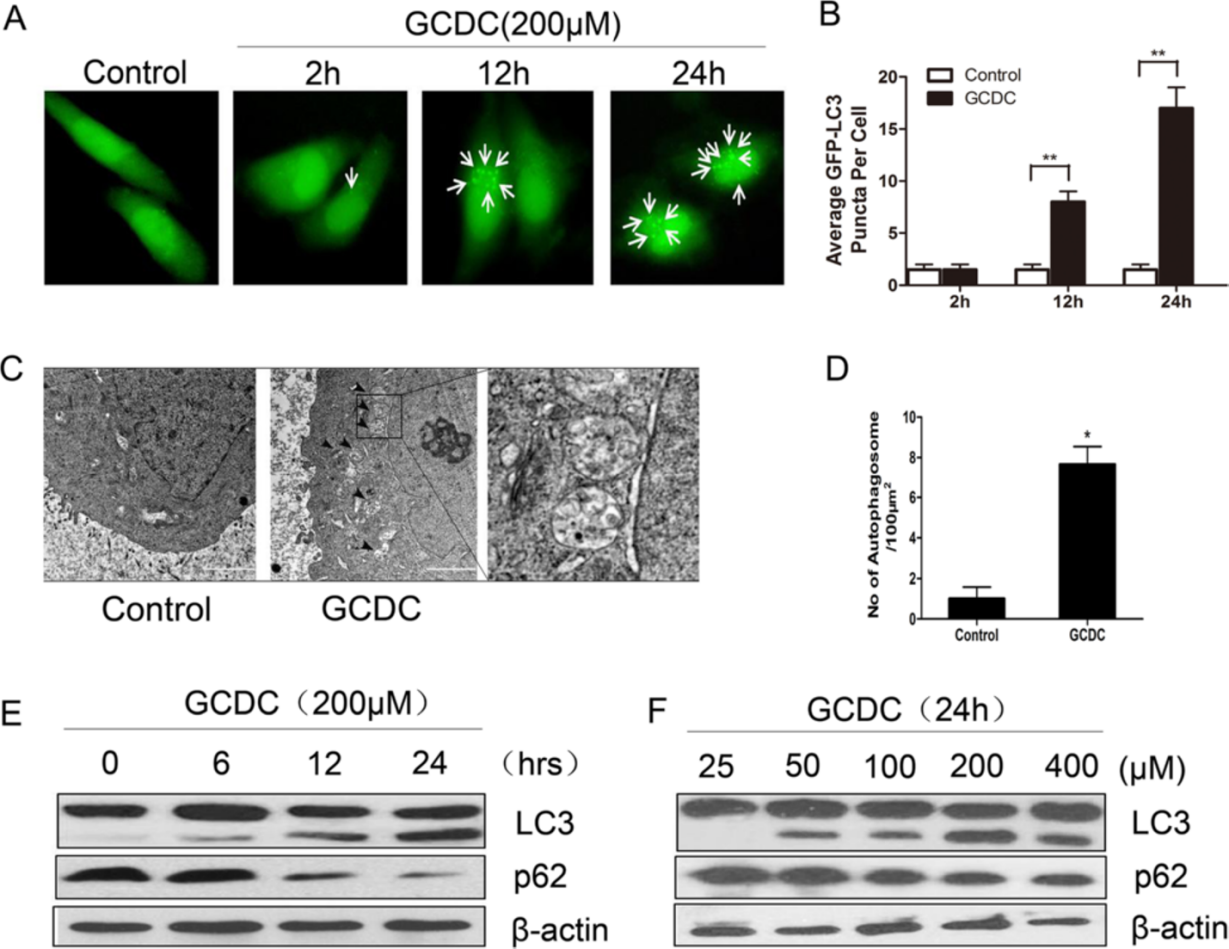
**Glycochenodeoxycholic acid induces autophagy in L02 hepatocytes. (A and B)** The L02 normal hepatocytes cells were transfected with GFP-tagged LC3 and then pretreated with GCDC (200 µM) as indicated (for 2, 12, and 24 hours). Images were taken under a fluorescence microscopy. GFP-LC3 puncta (mean ± SD) were quantified for each experiment. At least 20 cells were counted in each individual experiment. (**: P < 0.01). **(C)** Electron micrographs showing the cells ultrastructure as indicated. Black arrows indicate the autophagic vacuoles in the cytoplasm. (Bar: 20 µm). **(D)** The number of autophagosomes were quantified (mean ± SD) (*: P < 0.05) **(E)** Whole cell lysates were subjected to western blot to detect the expression of LC3 and p62 as indicated (for 0, 6, 12 and 24 hours). **(F)** LC3 and the p62 as indicated (for 25, 50, 100, 200, 400 µM) were examined by immunoblotting.

Since GCDC is the main toxic component of bile acid in cholestasis-induced injury, we treated L02 normal liver cells with GCDC to mimic cholestatic damage in our current study. The L02 cells were transfected with GFP-LC3 plasmid and analyzed by microscopy. Our results showed that GCDC treatment increased GFP-LC3 dots in the L02 liver cells in a time-dependent manner (Figure 2A and B). Electron microscopic analysis was then employed to observe the autopahgosomes. The results suggested the presence of characteristic double-membrane organelles in L02 cells pretreated with GCDC (Figure 2C and D). In addition, immunoblot analysis of endogenous LC3 (LC3-I to LC3-II) was markedly increased in a time- and dose- dependent manner upon GCDC stimulation, indicating a significantly increased formation and degradation in the group. Moreover, the amount of p62, a selective substrate of autophagy and an autophagy-related marker, was also markedly degraded given the GCDC in a time and dose study (Figure 2E and F). Taken together, these observations imply that GCDC acids could activate autophagy in hepatocytes in *vitro*.

### Autophagy protects against BDL-induced liver injury

**Figure 3:**
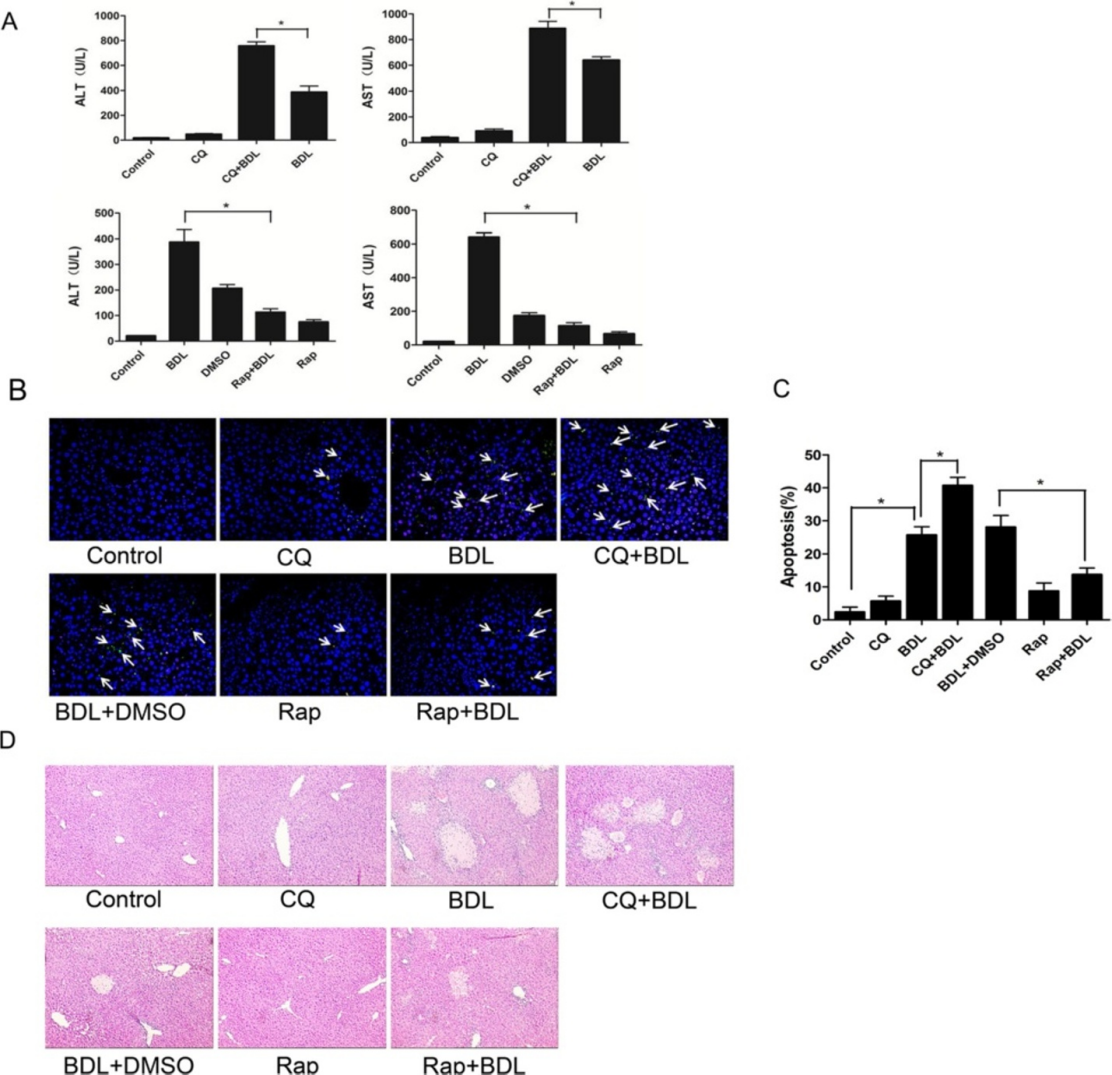
**Induction of autophagy protects against cholestatic injury in** ***vivo*** **.** Wild-type C57BL/6 mice were pretreated with Rapamycin or CQ intraperitoneally two or three times one week for two weeks, which was followed by BDL induction. Two weeks later, mice were sacrificed and were divided into seven groups: Control, CQ, BDL, CQ + BDL, Rap, BDL + DMSO, Rap + BDL. **(A)** Blood ALT and AST levels were examined and quantified in each group.(*: P < 0.05). **(B)** Apoptosis of different groups were determined by TUNEL assay and visualized by microscope. **(C)** The number of apoptosis (mean ± SD) was quantified from each group. More than 30 cells were counted in each experiment. (*: P < 0.05). **(D)** Representive photographs of H&E staining were presented. Original magnification: ×200.

Among the potential protective mechanisms, we hypothesized that activation of autophagy could play a role in limiting cholestasis-induced hepatotoxicity. Autophagic inhibitor-chloroquine (CQ) and inducer-rapamycin were applied. In our study, C57BL/6 mice were pretreated with rapamycin or CQ intraperitoneally two weeks earlier before BDL induction. As shown in Figure 3A, pretreatment of mice with CQ significantly elevated liver enzymes as measured by blood ALT and AST level, indicating that CQ addition greatly exacerbated BDL-induced liver injury. Meanwhile, treatment with rapamycin partly eliminated BDL-induced liver damage. CQ or rapamycin alone showed a slight shrinkage, suggesting that they had no significant effect on liver function. It is reported that cholestasis induced liver injury by increasing hepatocyte apoptosis, thus we examined the impact of autophagy on the survival of liver cell under BDL by TUNEL staining and H&E, respectively. As shown in Figure 3B, the mice of CQ + BDL group had higher percentage of the apoptotic cells (Tunnel-positive) compared to those of BDL group. However, co-treatment of rapamycin and BDL decreased the number of apoptotic hepatocytes (Figure 3B and C). Meanwhile, H&E staining were also performed to determine the liver damage. Both treatments of BDL and CQ + BDL had significant liver injury as focal necrosis was evident in livers; Necrosis increased following CQ + BDL while rapamycin treatment effectively reversed this rate. (Figure 3D). These results showed that autophagy inducer could partly resist cholestatic injury but inhibitor of autophagy aggravates it, which is consistent with our hypothesis that autophagy may be an important protector to cholestasis-induced liver injury. Taken together, these findings indicated that autophagy plays an active role in limiting BDL-induced cellular injury through reducing apoptosis or necrosis.

### Activation of autophagy reduced GCDC-induced hepatocyte apoptosis

**Figure 4:**
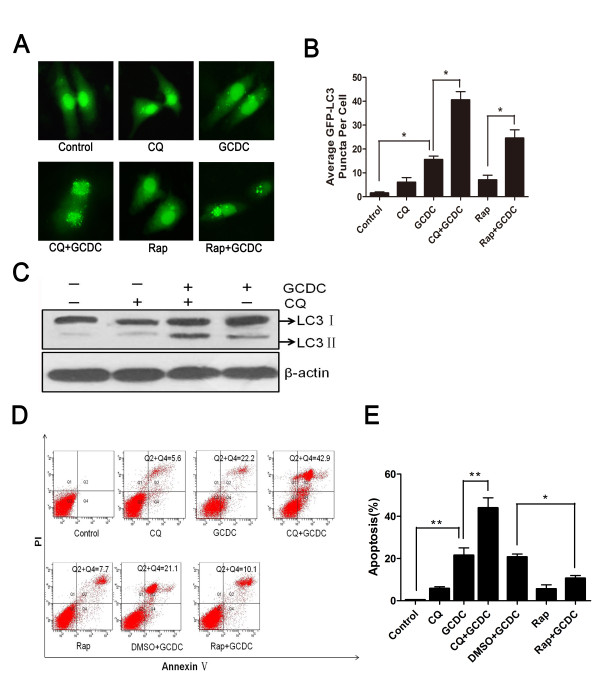
**Induction of autophagy protects against GCDC-induced cell apoptosis. (A)** L02 hepatocytes cells were transiently transfected with GFP-LC3 before treated with GCDC (200 µM), and then administered in the absence or presence of CQ (10 µM) or Rapamycin (2 µM) for 24 hours. **(B)** The percentage of cells with punctuate GFP-LC3 was measured; at least 30 cells were counted in each individual experiment. Data of at least three replicates were shown as (means ± SD) (*: P < 0.05). **(C)** In the absence or presence of CQ (10 µM), immunoblots were used to analyze endogenous LC3 expression, with Î²-actin as a loading control. **(D and E)** L02 hepatocytes were treated as indicated and the apoptosis was analyzed by flow cytometry using Annexin V-FITC. Data were shown individually as the (means ± SD) (*: P < 0.05, **: P < 0.01).

To further determine the role of autophagy on liver injury, in *vitro* studies were established. The autophagy inhibitor CQ and inducer rapamycin were used to examine the effects of autophagy in the injury of L02 cells by GCDC treatment. We found that pretreatment of L02 cells with CQ further increased the levels of GFP-LC3 puncta and LC3 form (Figure 4A,B and C), and treatment of hepatocytes with rapamycin confirmed the induction of autophagy induced by GCDC. It is with no doubt that autophagy was activated in response to GCDC. To investigate the effect of GCDC on cell death, the apoptosis of liver cells was examined by Annexin V-FITC assay. Futher, the PI staining rate (considered as reflecting necrotic cells) and the Annexin-V binding rate (reflecting apoptotic cells) were quantified, respectively. Figure 4D showed that both treatments of GCDC and CQ + GCDC caused obvious elevated necrosis in L02 cells; however, there was no significant difference between them (data not shown). On the contrary, the percentage of apoptotic cells in the group of CQ + GCDC was significantly higher than that of GCDC alone. There were only 22.2% and 42.9% apoptotic cells in GCDC group and CQ + GCDC group, respectively. Meanwhile, the percentage of apoptosis was 21.1% in the DMSO + GCDC group, while it reduced to 10.1% in the Rap + GCDC group (Figure 4E). Compared with GCDC alone, necrosis in CQ and GCDC combined treatment group showed no significant difference but apoptosis was much higher, suggesting that GCDC induced apoptosis, but not necrosis caused by hepatic injury of GCDC. Thus, our data imply that induction of autophagy protects against GCDC-induced apoptosis, but not necrosis. These results further confirm that autophagy prevents hepatocytes damage, and that rapamycin preserves liver death, at least partly, by enhancing autophagy.

### ROS contributes to cholestasis-induced liver injury

**Figure 5:**
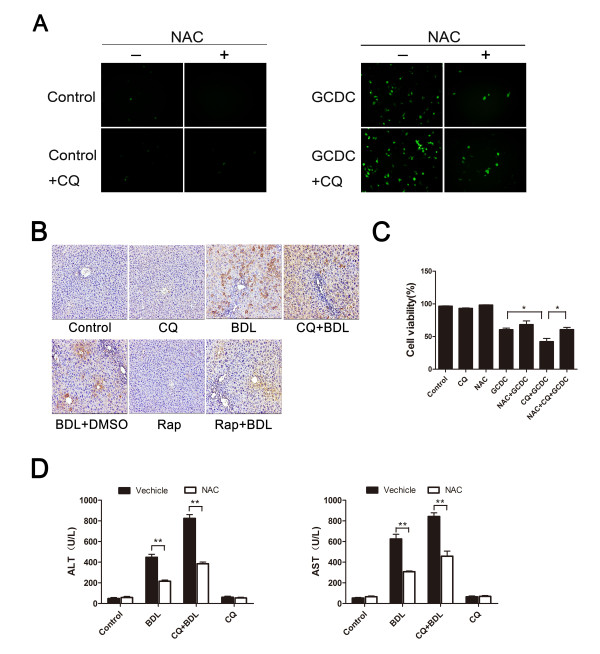
**Autophagy protects hepatocytes from cholestatic injury by eliminating ROS. (A)** L02 normal liver cells were incubated with GCDC for 24 h with 10 µM CQ. Cellular ROS activity was determined using DCF-DA staining. **(B)** Immunohistochemical staining of 8-OhdG in the liver of mice. **(C)** L02 normal liver cells were pretreated with 10 mM NAC for 2 h, and cells were incubated in GCDC (200 µM) or with 10 µM CQ for the indicated time. Cell viability was assessed by MTT assay. Data (mean ± SD) were determined in at least three independent experiments. (*: P < 0.05). **(D)** Mice were treated as indicated and serum levels of ALT and AST were determined. Data were expressed as mean ± SD. (**: P < 0.01).

GCDC has been known as an inducer of oxidative stress, and increased ROS lead to hepatocyte damage [22, 23]. To determine whether ROS is involved in GCDC induced by cholestasis, we examined the role of DCF, a measure of hydroperoxide generation for ROS. As shown in Figure 5A, intracellular levels of ROS were detected. ROS expression was increased when treated with GCDC, while there were marked increased in cells with CQ combination. At the same time, we also explored whether the generation of ROS was an important event in *vivo*. 8-Hydroxy-2’deoxy Guanosine (8-OHdG), an indicator of DNA oxidative damage, in liver tissue of mice was examined by immunostaining [24]. In Figure 5B, massive positive immuno-reactivity of 8-OHdG was found in BDL-treated liver, and much stronger intensity was observed after co-treatment with CQ. Tretreatment of rapamycin showed weak expression, suggesting that autophagy contributes to minimize the accumulation of ROS (Figure 5A and B). To evaluate whether enhanced ROS levels may contribute to the cell death of hepatocytes induced by cholestasis, we applied the N-acety-L- cysteine (NAC) to eliminate ROS. As shown in Figure 5C, pre-incubation with ROS scavenger NAC significantly alleviated cell death upon GCDC treatment. It can be found that NAC prominently promoted the survival of L02 cells in the CQ + GCDC group. Furthermore, liver injury after NAC treatment determined by ALT and AST level is shown in Figure 5D. Not surprisingly, the ALT and AST levels in serum were significantly reduced in CQ + BDL group after inhibiting ROS by NAC. This finding indicated that ROS is involved in the hepatocytes cytotoxic induced by cholestasis. Therefore, we conclude that autophagy protected hepatocytes from cholestatic injury by decreasing ROS activity.

## Discussion

The major finding of this study is that autophagy is a survival mechanism for liver to protect against cholestasis-induced hepatocyte injury. Our results showed that blockade of autophagy by autophagic inhibitor-CQ exacerbated cholestasis-induced liver injury and hepatocytes death. In contrast, inducer of autophagy by rapamycin could partly inhibit the cholestasis hepatotoxicity. Moreover, modulation of ROS by autophagy could contribute to the cell survival during cholestasis.

Autophagy is an essential component occurs at low basal level in most cells to perform homeostasis functions such as protein and organelle tunerover, and be upregulated when response to some stressors. Activation of autophagy has been reported with liver injury induced by steatosis, alcoholic consumption, ischemia/reperfusion and toxic drugs [19, 25–27]. For example, in liver ischemia reperfusion injury, autophagy mainly has a prosurvival activity, allowing the cell for coping with nutrient starvation and anoxia [28, 29]. However, using animal models to study whether autophagy is involved in and its function has not been reported in this particular condition induced by cholestasis. We suspect that the enhancing autophagic capacity of the liver may adapt to the change of cholestatic environment and serve as an effective survival strategy to clearance of abnormal or damaged hepatic proteins and organelles. In our study, we found that autophagy was activated when liver cells explore to GCDC or BDL in *vitro* or in *vivo* at first. Then the result from the serum and cell morphology showed that treatment with CQ would exacerbated the hepatic injury by increasing apoptosis in cholestasis. Conversely, administration of the rapamycin significantly promoted the liver survival. Our data suggest that autophagy help hepatocyte overcoming cholestatic injury. Similarly, the protective effect of autophagy restores sevoflurane preconditioning lost by longer ischemic [30] and APAP induced hepatotoxicity [19]. These findings indicate that autophagy activation in cholestatic injury directly contributes to the survival of liver cells.

How autophagy promotes cell survival to cholestasis environment, however, remains to be explored. Detection of apoptosis and necrosis are further conducted in liver tissue and hepatocytes. Apoptosis has been observed to occur with necrosis following BDL, but in *vitro* only apoptosis is to present the major cell death treated with GCDC. There may be two reasons for this phenomenon. First, actual bile salt concentration contacted in liver is not entirely consistently in *vivo* and in *vitro*. Second, the liver injury induced by cholestasis includes many factors, such as oxidative stress, mitochondrial damage, and cell membrane disruption and so on. Therefore, the different environment in *vivo* or in *vitro* results in different stimulation effects on bile acids effects. Our findings showed that there was no difference in necrosis but apotosis between the CQ and CQ + GCDC group. All of these results supported our hypothesis that pretreated with CQ exacerbated the hepatic injury due to increased apoptosis in cholestasis. Conversely, rapamycin administration would have the opposite effect, restoring the cell death. Previous report has been shown that autophagy, apoptosis and necrosis can overlap and autophagy delays the onset of both apoptotic and necrotic cell death in a model of ischemic cell death [31].

ROS was shown to act as a critical signal in the pathogenesis of bile acid-induced hepatocyte injury [23]. In our study, we also found that cholestasis-induced autophagic activation, at least partially, via oxidative stress, which is critical for liver cells survival. We have observed a modest increase in cellular ROS activity caused by GCDC or BDL with model of cholestasis. Combination of CQ and GCDC or BDL resulted in a marked increase in ROS generation while surpression of ROS generation was shown when pretreated with rapamycin. More importantly, increased levels of ROS contributed to cell death and liver damage in cholestatic liver cells and tissues when autophagy was inhibited, but treatment with the antioxidant NAC antioxidant markedly reduced this phenonemon. Consisting with these reports, Ding ZB *et al.* found oxaliplatin-induced ROS generation is augmented by autophagy inhibition and has an important role in cell death [32]. Sun K *et al.* stated that induction of autophagy reducing ROS-induced necrosis to suppressed ischemic liver injury [27]. Our results support that autophagy is a cellular self-defense response to alleviate cholestasis-induced liver injury through modulating oxidative stress. However, current study has suggested that the mitophagy is the elimination of an important source of ROS [33]. Thus, it is conceivable that autophagic degradation of damaged mitochondria is a part of the protection mechanism against cholestatic liver injury. Our work, however, does not clarify the specific mechanisms via which autophagy modulates ROS, especially the role of mitophagy in the cholestasis induced hepatocytes injury. Further studies are warranted in future.

In this study, we found that activation of autophagy can enhance the survival of liver injury induced by cholestasis. A proper autophagy capability in the liver may be crucial to reduce the detrimental effects of cholestasis, and that enhancement of autophagy may be a possible therapeutic strategy to mitigate the pathology associated with cholestasis liver disease.

## Methods

### Animals model

Male C57BL/6 mice, 6–8 weeks old, weighting 20-25 g, were purchased from the Shanghai Experimental Animal Center of the Chinese Academy of Sciences, Shanghai, China. All mice used in this study were housed in pathogen-free conditions, and all procedures were performed in accordance with guidelines established by the Chinese Academy of Sciences’ Committee on Animals. The animals received the laparotomy with bile duct ligaltion were performed as previously described in detail [34]. In the control group, mice had undergone the surgical procedures but without BDL. Induction or suppression of autophagy was achieved by intraperitoneal administration of chloroquine (60 mg/kg), rapamycin (2 mg/kg) unless otherwise indicated in figure legends.

### Regents

Chloroquine (CQ), Rapamycin, Glycochenodeoxycholate (GCDC) and N-acetyl-cysteine (NAC) were purchased from Sigma-Aldrich (St Louis, MO).

### Transient transfection and identification of autophagy

GFP-tagged LC3 expression vector has been utilized to demonstrate the occurrence of autophagy and was detected using an inverted fluorescence microscope. L02 cells were seeded (1 × 10^3^cells/well) in 96-well plates overnight and were transiently transfected with GFP-LC3 expressing plasmids using Fugene HD transfection reagent (Calbiochem, La Jolla, CA) according to the manufacturer’s instructions. After initial treatment, autophagy was detected by counting the number of GFP-LC3-positive dots per cell under fluorescence microscope (Olympus IX71).

### Measurement of liver function

Two weeks after BDL, blood samples were collected from all mice. The plasma alanine aminotransferase (ALT), aspartate aminotransferase (AST) and totalbilirubin (TBIL) levels were tested with a biochemical autoanalyzer (Fuji Medical System, Tokyo, Japan) according to the manufacturer’s instructions.

### Western blot analysis

After special treatment, cells and tissues were lysed in RIPA lysis buffer (Beyotime) with 1 mM PMSF. Equal amount of protein was separated by SDS-PAGE and transferred to NC membrane. The membranes were washed, blocked, and incubated with specific primary anti-human antibodies against LC3 (Novus Biologicals, Littleton, CO) and p62/SQTM (Cell signaling Technology, Beverly, MA), Î²-actin antibody (Hangzhou HuaAn Biotech, Zhejiang, China), followed by incubation with horseradish peroxidase-conjugated secondary antibodies (Hangzhou HuaAn Biotech). Signals were visualized by chemiluminescent detection (Beyotime).

### Histological examination and tunnel staining

Liver tissues were fixed in 4% paraformaldehyde, sectioned, and mounted on glass slides then stained with Meyer’s hematoxylin and eosin. Each sample was observed at a 200× magnification of microscopic field in 5 randomly selected areas. Tunnel staining (Calbiochem, La Jolla, CA) was used to assess the apoptosis level of paraffin-embedded fraction slides, according to the manufacturer’s instructions. The number of positive-apoptotic cells were counted and expressed to the total number of cells within 5 random fields (200×) of cells.

### Electron microscopic analysis

At room temperature cells were fixed in 2.5% glutaraldehyde in PBS (pH = 7.4) for two hours, postfixed in 1% osmium tetroxide in water for one hour. After dehydrating in an ascending series of ethanol, the samples were then embedded in araldite. 50-60 nm sections were cut on a LKB-I ultramicrotome and picked up on copper grids, post-stained with uranyl acetate and lead citrate, observed in a Philips CM-120 TEM.

### Flow cytometric analysis

Annexin V-fluorescein isothiocyannate (FITC) assay was used to measure cell death by flow cytometry according to the manufacturer’s instructions (Nanjing Keygen Biotech, China). Briefly, cells were collected together and resuspended in 300 µl 1 × binding buffer containing 5 µl of Annexin V and 5µof PI for 30 min at room temperature in the dark. After incubation, samples were analyzed by a BD FACSAria flow cytometer within one hour.

### Measurement of intracellular ROS level

To examined the accumulation of reactive oxygen species (ROS), cells were incubated with 10 µM 2’, 7’-dichlorofluorescein diacetate (DCF-DA; Invitrogen) for 30 min at 37°C, respectively, followed by fluorescence microscopy.

### Statistical analysis

All of the experiments were repeated at least three times. Quantitative data were expressed as mean ± SD. Significance between two groups was performed with the Student’s *t* test. P < 0.05 was considered statistically significant. Statistical analysis was performed with GraphPad Prism 5.0 software.

## Abbreviations

ALT: Alanine aminotransferase
Annexin V-FITC: Annexin V -fluoresceinisothiocyannate
AST: Aspartateaminotransferase
BDL: Bile duct ligation
CQ: Chloroquine
DAB: Diaminobenzidine
DMSO: Dimethyl Sulfoxide
EM: Electron microscope
GCDC: Glycochenodeoxycholate
GFP: Green Fluorescent Protein
H&E: Hematoxylin and eosin
HRP: Horseradish Peroxidase
LC3: Microtubule-associated protein 1 light chain 3
MTT: Methyl thiazolyl tetrazolium
NAC: N-acetyl-l-cysteine
8-OhdG: 8-hydroxy-2’ –deoxyguanosine
p62: Sequestosome 1
PAGE: Polyacrylamide gel electrophoresis
PMSF: Phenylmethyl sulfonyl fluoride
RAP: Rapamycin
ROS: Reactive oxygen species
TBIL: Total bilirubin
TUNEL: Terminal deoxynucleotidyl transferase-mediated dUTP nick-end labeling.

## References

[1] Guicciardi ME, Gores GJ (2002). Bile acid-mediated hepatocyte apoptosis and cholestatic liver disease. Dig Liver Dis.

[2] Reichel C, Meier-Abt PJ (1997). Cholestatic liver diseases. Ther Umsch.

[3] Karvonen J, Kairisto V, Gronroos JM (2006). Stone or stricture as a cause of extrahepatic cholestasis–do liver function tests predict the diagnosis?. Clin Chem Lab Med.

[4] Zhangxue H, Min G, Jinning Z, Yuan S, Li W, Huapei S, Rui L, Chunyu Z (2012). Glycochenodeoxycholate induces rat alveolar epithelial type II cell death and inhibits surfactant secretion in *vitro*. Free Radic Biol Med.

[5] Heuman DM, Mills AS, McCall J, Hylemon PB, Pandak WM, Vlahcevic ZR (1991). Conjugates of ursodeoxycholate protect against cholestasis and hepatocellular necrosis caused by more hydrophobic bile salts. In vivo studies in the rat. Gastroenterology.

[6] Schmucker DL, Ohta M, Kanai S, Sato Y, Kitani K (1990). Hepatic injury induced by bile salts: correlation between biochemical and morphological events. Hepatology.

[7] Schoemaker MH, Gommans WM, de la Conde Rosa L, Homan M, Klok P, Trautwein C, van Goor H, Poelstra K, Haisma HJ, Jansen PL, Moshage H (2003). Resistance of rat hepatocytes against bile acid-induced apoptosis in cholestatic liver injury is due to nuclear factor-kappa B activation. J Hepatol.

[8] Patel T, Bronk SF, Gores GJ (1994). Increases of intracellular magnesium promote glycodeoxycholate-induced apoptosis in rat hepatocytes. J Clin Invest.

[9] Czaja MJ (2011). Functions of autophagy in hepatic and pancreatic physiology and disease. Gastroenterology.

[10] Yorimitsu T, Klionsky DJ (2005). Autophagy: molecular machinery for self-eating. Cell Death Differ.

[11] Moreau K, Luo S, Rubinsztein DC (2010). Cytoprotective roles for autophagy. Curr Opin Cell Biol.

[12] Kuma A, Matsui M, Mizushima N (2007). LC3, an autophagosome marker, can be incorporated into protein aggregates independent of autophagy: caution in the interpretation of LC3 localization. Autophagy.

[13] Mathew R, Karp CM, Beaudoin B, Vuong N, Chen G, Chen HY, Bray K, Reddy A, Bhanot G, Gelinas C, Dipaola RS, Karantza-Wadsworth V, White E (2009). Autophagy suppresses tumorigenesis through elimination of p62. Cell.

[14] Narendra D, Kane LA, Hauser DN, Fearnley IM, Youle RJ (2010). p62/SQSTM1 is required for Parkin-induced mitochondrial clustering but not mitophagy; VDAC1 is dispensable for both. Autophagy.

[15] Manley S, Williams JA, Ding WX (2013). Role of p62/SQSTM1 in liver physiology and pathogenesis. Exp Biol Med (Maywood).

[16] Arroyo DS, Gaviglio EA, Peralta Ramos JM, Bussi C, Rodriguez-Galan MC, Iribarren P (2014). Autophagy in inflammation, infection, neurodegeneration and cancer. Int Immunopharmacol.

[17] Ding WX, Li M, Chen X, Ni HM, Lin CW, Gao W, Lu B, Stolz DB, Clemens DL, Yin XM (2010). Autophagy reduces acute ethanol-induced hepatotoxicity and steatosis in mice. Gastroenterology.

[18] Igusa Y, Yamashina S, Izumi K, Inami Y, Fukada H, Komatsu M, Tanaka K, Ikejima K, Watanabe S (2012). Loss of autophagy promotes murine acetaminophen hepatotoxicity. J Gastroenterol.

[19] Ni HM, Bockus A, Boggess N, Jaeschke H, Ding WX (2012). Activation of autophagy protects against acetaminophen-induced hepatotoxicity. Hepatology.

[20] Singh R, Kaushik S, Wang Y, Xiang Y, Novak I, Komatsu M, Tanaka K, Cuervo AM, Czaja MJ (2009). Autophagy regulates lipid metabolism. Nature.

[21] Kabeya Y, Mizushima N, Ueno T, Yamamoto A, Kirisako T, Noda T, Kominami E, Ohsumi Y, Yoshimori T (2000). LC3, a mammalian homologue of yeast Apg8p, is localized in autophagosome membranes after processing. EMBO J.

[22] Yerushalmi B, Dahl R, Devereaux MW, Gumpricht E, Sokol RJ (2001). Bile acid-induced rat hepatocyte apoptosis is inhibited by antioxidants and blockers of the mitochondrial permeability transition. Hepatology.

[23] Sokol RJ, Winklhofer-Roob BM, Devereaux MW, McKim JM (1995). Generation of hydroperoxides in isolated rat hepatocytes and hepatic mitochondria exposed to hydrophobic bile acids. Gastroenterology.

[24] Maes M, Mihaylova I, Kubera M, Uytterhoeven M, Vrydags N, Bosmans E (2009). Increased 8-hydroxy-deoxyguanosine, a marker of oxidative damage to DNA, in major depression and myalgic encephalomyelitis/chronic fatigue syndrome. Neuro Endocrinol Lett.

[25] Lin CW, Zhang H, Li M, Xiong X, Chen X, Dong XC, Yin XM (2013). Pharmacological promotion of autophagy alleviates steatosis and injury in alcoholic and non-alcoholic fatty liver conditions in mice. J Hepatol.

[26] Osna NA, Thomes PG, Jr TM (2011). Involvement of autophagy in alcoholic liver injury and hepatitis C pathogenesis. World J Gastroenterol.

[27] Sun K, Xie X, Liu Y, Han Z, Zhao X, Cai N, Zhang S, Song J, Wei L (2013). Autophagy lessens ischemic liver injury by reducing oxidative damage. Cell Biosci.

[28] Yun N, Cho HI, Lee SM (2014). Impaired autophagy contributes to hepatocellular damage during ischemia/reperfusion: heme oxygenase-1 as a possible regulator. Free Radic Biol Med.

[29] Rautou PE, Mansouri A, Lebrec D, Durand F, Valla D, Moreau R (2010). Autophagy in liver diseases. J Hepatol.

[30] Shiomi M, Miyamae M, Takemura G, Kaneda K, Inamura Y, Onishi A, Koshinuma S, Momota Y, Minami T, Figueredo VM (2014). Induction of autophagy restores the loss of sevoflurane cardiac preconditioning seen with prolonged ischemic insult. Eur J Pharmacol.

[31] Loos B, Genade S, Ellis B, Lochner A, Engelbrecht AM (2011). At the core of survival: autophagy delays the onset of both apoptotic and necrotic cell death in a model of ischemic cell injury. Exp Cell Res.

[32] Ding ZB, Hui B, Shi YH, Zhou J, Peng YF, Gu CY, Yang H, Shi GM, Ke AW, Wang XY, Song K, Dai Z, Shen YH, Fan J (2011). Autophagy activation in hepatocellular carcinoma contributes to the tolerance of oxaliplatin via reactive oxygen species modulation. Clin Cancer Res.

[33] Kanki T (2010). Nix, a receptor protein for mitophagy in mammals. Autophagy.

[34] Tsai LY, Lee KT, Liu TZ (1998). Evidence for accelerated generation of hydroxyl radicals in experimental obstructive jaundice of rats. Free Radic Biol Med.

